# Improved quality control and moisture determination in dry kava (*Piper methysticum*) from Vanuatu: a practical oven-drying approach

**DOI:** 10.7717/peerj.21102

**Published:** 2026-07-07

**Authors:** Daniel Tari, Hancy Tabi, Jacinta Botleng, Rolina Kelep, Ladyshia Regenvanu, Cassandra Tangaras, Galana Siro, Krishna K. Kotra, Atanas Pipite, Lindon Havimana, Ronick S. Shadrack

**Affiliations:** 1 Laboratory, Vanuatu Bureau of Standards, Port Vila, Vanuatu; 2 School of Agriculture, Geography, Environment, Ocean and Natural Sciences (SAGEONS), The University of the South Pacific, Emalus Campus, Shefa, Port Vila, Vanuatu; 3 Department of Fisheries Studies, Faculty of Agriculture, Fisheries and Forestry, Solomon Island National University, Honiara, Solomon Islands

**Keywords:** Kava quality, Moisture content, FTIR, Colorimetric absorbance, IR moisture analyzer, Dry kava, Oven-drying

## Abstract

Dry kava (*Piper methysticum*) exports in the Pacific Island region are booming, but stringent quality control remains a critical factor in maintaining the reputation and competitiveness of these exports in international markets. This study evaluates trends in the quality of dry kava export in Vanuatu, develops a practical Association of Official Analytical Chemists (AOAC) oven-drying method for moisture analysis, and assesses the effects of drying on dried kava samples and colorimetric absorbance. A national database of colorimetric absorbance measurements from 2016 to 2024 was analysed, using the benchmark that high-quality “noble” kava has a maximum absorbance threshold value of 0.84 ± 0.05, according to previous studies. For moisture determination, kava samples were dried at 105 °C in an oven for 0.5 h, 1 h, 1.5 h, and 2 h, and compared against the infrared (IR) thermal combustion method, with further verification by Fourier Transform Infrared (FTIR) spectral analysis. Results indicate that kava quality in Vanuatu has significantly improved, with absorbance values decreasing from 0.72 to 0.48 (2016 to 2024), indicating stronger quality control across the export chain. The 2 h oven drying method closely matched the IR method with high agreement (R-Square (R2) = 0.99, Bias = 0.12%, Precision = 0.2%, Root Mean Square Error (RMSE) = 0.20). It also demonstrated better repeatability (Relative Standard Deviation (RSD) = 0.22%) and strong reproducibility (RSD = 0.29%). The FTIR analysis indicated minimal spectral variation among samples dried for 2 h, suggesting that only limited chemical modifications occurred during this drying stage. In contrast, pronounced spectral shifts were observed at 1,050, 1,640, 1,740, and 2,920 cm^1^ after 4 h of drying, reflecting substantial alterations in the chemical composition of the kava samples. The samples achieved a constant weight after 4 h of drying at 125 °C and 131 °C, exhibiting significantly greater weight loss compared to those dried at 105 °C for the same duration. Furthermore, a significant correlation (*p* ≤ 0.05) between colorimetric absorbance and weight loss was observed, indicating that moisture content strongly influences optical properties thereby supporting more accurate nobility assessment. These findings demonstrate that the 2 h oven drying method at 105 °C is a practical and reliable approach for routine moisture analysis in dry kava and can support improved quality control in the kava industry.

## Introduction

Kava is a vernacular name derived from Tongan and refers to a traditional plant-based beverage prepared from a botanical plant species belonging to the pepper family (Piperaceae) and is commonly known as ‘awa in Hawaii, yaqona in Fiji, and malok in Vanuatu (Bian et al., 2020). Scientifically it is known as *Piper methysticum*, meaning “intoxicating pepper”, which correlates to its psychoactive effects. Native to Melanesian, Polynesia and Micronesia, kava holds cultural significance across these regions. It is highly valued for its properties and its integral role in traditional ceremonies and social rituals within these communities (Bian et al., 2020; Celentano et al., 2019; Norton & Ruze, 1994). Kava beverage is traditionally prepared through cold water (22 °C to 28 °C) extraction of the ground fresh or dried roots and peeled basal stumps of the plant (Lebot & Legendre, 2016). The active constituents of kava linked to its sedative and anxiolytic properties primarily consist of kavalactones. In addition, kava contains other biological relevant compounds, including alkaloids and chalcones, notably flavokavains A, B, and C (Lebot, Kaoh & Legendre, 2020). Kava quality is largely determined by its chemotype which influences the physiological effects, as well as the total kavalactone content, which correlates with the intensity of these effects (Lebot, Do & Legendre, 2014).

In Vanuatu, kava varieties are categorized into “nobles,” “tudei,” and “wichmannii” based on their physiological effects, which are largely influenced by the levels of six major kavalactones (kavain, methysticin, dihydromethysticin, dihydrokavain, yangonin, and desmethoxyyangonin). High-Performance Liquid Chromatography (HPLC) profiling of *Piper methysticum* distinguishes noble from tudei (non-noble) kava based on characteristic kavalactone and flavokavain patterns. Noble cultivars display chemo-types such as 245, 246, 245,631, and 426,351, dominated by kavain and dihydrokavain, while tudei cultivars exhibit 652,431 and 531,246, rich in dihydromethysticin and methysticin (Lebot & Levesque, 1996). Additionally, flavokavain B (FKB) concentration serves as a quality marker, with FKB <0.2% (w/w) indicating noble kava, and levels ≥ 0.2% (w/w) indicating tudei or lower-grade types (Lebot & Legendre, 2016; Lebot & Levesque, 1996; Showman et al., 2015; Teschke, Sarris & Lebot, 2011). Using kava lactone and flavokavain rational, the colorimetric method was established as low cost qualitative method suitable for routine check prior to export of kava (Lebot & Legendre, 2016).

Advanced analytical techniques, such as HPLC and Gas Chromatography-Mass Spectrometry (GC-MS), offer precise quantification of kavalactones (Lebot, Michalet & Legendre, 2019). However, these methods are often cost-prohibitive and require sophisticated laboratory infrastructure, making them impractical for routine use in many small island developing states (SIDS). Recent advances in differentiating noble kava and tudei day kava using genetic finger printing and Fourier Transform Infrared spectroscopy (FTIR) are promising (Lebot et al., 2024), however, access and costs limit routine application of quality testing. The colorimetric test has emerged as a practical alternative for assessing kava quality. This analytic test involves obtaining extracts from dried kava (roots and chips) powder samples with a solvent (commonly acetone) and measuring the absorbance at a specific wavelength (440 nm) with colorimeter. At 440 nm, the mean absorbance of acetonic extracts from different kava varieties serve as a comparative metric or indicator. Specifically, the mean absorbance values are ≤ 0.42 for noble varieties, ≥ 0.84 for tudei varieties, and ≥ 1.30 for wichmannii varieties (Lebot & Legendre, 2016). The absorbance values correlate with the concentration of kavalactones and flavokavains, which are more efficiently extracted in solvents like acetone than in water, allowing differentiation between noble and non-noble kava varieties (Lebot, Kaoh & Legendre, 2020; Lebot & Legendre, 2016).

The colorimetric test method is widely employed in countries like Fiji and Hawaii due to its affordability and simplicity (Lebot, 2016; Lebot & Legendre, 2016). Pursuant to the Codex Regional Standard for Kava Products for Use as a Beverage (CXS 336R-2020), kava products shall meet the criterion “absorbance of acetone extracts (≤ 0.9 absorbance units at 400 nm)” to confirm a Noble variety (Codex Alimentarius Commission, 2020); however, Vanuatu possesses the most diverse varieties of noble and tudei cultivars (Lhuissier et al., 2017), necessitating stricter quality control due to the high variation in colorimetric absorbance values. In this study, a threshold of ≤ 0.84 absorbance units at 440 nm was applied to classify samples as noble, while those above this value were classified as tudei, thereby aligning with both the regional standard (Codex Alimentarius Commission, 2020) and empirical differentiation of cultivar groups.

The colorimetric test is limited by sample moisture, which can affect absorbance and classification accuracy (Lhuissier et al., 2017). For instance, samples with elevated moisture content can display reduced absorbance intensities during colorimetric analysis, thereby increasing the risk of erroneously classifying non-noble kava as noble. Studies have highlighted the need to account for moisture content to enhance the accuracy of the colorimetric test (Lhuissier et al., 2017). To address these limitations, establishing a standardized method for determining moisture content in kava is essential.

The Association of Official Analytical Chemists (AOAC) recommends oven-drying samples at 105 °C to determine moisture content in food products. Studies have shown that drying food-related products at 105 °C for specific durations (*e.g.*, 2 h and 3 h) yield reliable results, although drying time may vary depending on the product’s matrix and composition (Ahn et al., 2014). In this study, a drying temperature of 105 °C is chosen with exposure time ranging from 0.5 h to 2 h to identify the optimal condition to maintain drying efficiency and preserve bioactive compounds in kava. Similar work done by Ahn et al. (2014) confirmed that drying sensitive matrices at approximately 2 h gives accurate moisture measurements, lessening the risk of over-estimating moisture as a result of degraded volatile compounds. Thus, kava’s unique composition may require modifications to AOAC protocol to accurately measure moisture content while preventing the degradation of heat-sensitive compounds (Teschke, Sarris & Lebot, 2011). Moisture content of dried kava was determined with the oven-drying method at 105 °C to constant weight, as recommended by Codex and national standards (Lebot, Kaoh & Legendre, 2020). Previous studies have also employed thermogravimetric analysis (TGA) and Karl Fischer titration to quantify residual water in kava powders and rootstocks (Aijaz et al., 2024; Lebot, 2016).

For dried kava quality assessment, dried kava (grounded basal stem and roots), whether oven-dried or sun-shade dried, should ideally have an average moisture content of 12% or lower to prolong its shelf life and limit biochemical degradation (Singh, 2004). When kava is over dried, several important changes can occur that comprise its quality (Teschke, Sarris & Lebot, 2011). Kavalactones, are sensitive to heat, air, and light (Bilia et al., 2004). The natural yellow-brown color of kava is primarily due to polyphenols and other plant pigments, rather than flavokavains B. Prolonged drying or storage can lead to oxidation of these compounds, resulting in fading or dulling of the color, which is often related to loss of freshness and reduced perceived quality (Lebot, Merlin & Lindstrom, 1997; Sarris, LaPorte & Schweitzer, 2011). Over drying diminishes root permeability, impairing solvent penetration and yielding less potent beverages. Hence, beverages made from over-dried kava may exhibit reduced calming effects due to lower kavalactone content (Sarris, LaPorte & Schweitzer, 2011). The objectives of the present study are: (i) to examine the trend in Vanuatu kava quality from 2016 to 2024 (period in which kava quality check was introduced) *via* calorimetric absorbance, with moisture content unstandardized and, (ii) to establish and validate a standardized oven-drying protocol for kava moisture analysis, with subsequent absorbance correction for moisture content. We hypothesized that drying samples at a standardized temperature of 105 °C would improve the consistency and reliability of moisture content determination across samples.

## Methods

### Sampling and quality control of dry kava export

The *Piper methysticum* samples representing distinct cultivars from major kava-producing islands in Vanuatu were selected from registered growers and verified source to capture variability in origin and processing. Each sample was authenticated by botanists from the Vanuatu Department of Agriculture using morphological characteristics consistent with noble kava varieties, such as stem color, internode structure, and leaf shape, following the classification described by Lebot & Lévesque (1989). Only confirmed *P. methysticum* plants were used in the analysis. The kava samples (roots, chips and powder), were collected by the Vanuatu Biosecurity (VB) officers during routine controls of exported kava and delivered to the Vanuatu Bureau of Standards (VBS) laboratory for quality analysis at room temperature, covering the years 2016 to 2018 (*n* = 1,097), 2019 (*n* = 457), 2020 (*n* = 366), 2021 (*n* = 208), 2022 (*n* = 191), 2023 (*n* = 232) and 2024 (*n* = 287). The analysis conducted is based on the data obtained from these samples within that timeframe. Although 2022 has the smallest sample size, the primary objective of the analysis is to assess the quality of the export samples rather than to compare production volumes across years. Samples were submitted by exporters for routine export quality testing. Sample origin and form (root, chips, or powder) were not controlled by the laboratory and depended on exporter submissions. Sampling across years reflects submission frequency rather than a structured annual design. Irrespective of sample types, tudei and wild (wichmannii) kava always have high absorbance above the kava nobility threshold. Validation sample size was limited by routine export submissions, and uncertainty is accounted for through the reported standard error.

The chemical solvent, primarily acetone, was purchased from South Pacific Suppliers Ltd., Port Vila. A colorimeter (WPA MODEL C07500) was used for absorbance measurements of the kava extract. Kava testing was conducted according to the method of Lebot & Legendre (2016). Samples of pulverized kava powder (<two mm) were thoroughly mixed with acetone by inverting tubes repeatedly for 1 min, followed by homogenization using an ultrasonic cleaner water bath for 30 mins and left overnight (16 h). The solvent extracts were separated by centrifugation at 3,000 rpm (1,510×g) using a rotor radius of 15 cm for 10 min and the supernatants were employed for colorimetric determinations at 440 nm. The kava quality criteria were determined by measuring the absorbance of noble and non-noble kava varieties as reported in Lebot & Legendre (2016). The absorbance readings of kava-acetone extracts above the nobility threshold of 0.84 is categorized as tudei and is banned for export. Whereas, noble kava varieties exhibit absorbance values from 0.12 to 0.84, making it suitable for export.

### Moisture method and validation

A total of 10 bags (2  kg per bag) of dried kava comprising five bags of whole roots and five bags of chips were randomly sourced from South Seas Exporters. The validation sample size (10 bags, *n* = 30 in triplicate) was sufficient to assess analytical repeatability for routine export quality testing; however, we acknowledge that a larger sample size would further strengthen method robustness. The morphological characteristics of kava were determined according to standard descriptions outlined in the American Herbal Pharmacopoeia (Pharmacopoeia, 2025), when sourcing the samples. Each sample was analyzed in triplicate (*n* = 30) to ensure data consistency. Although limited in number, this sampling size aligns with preliminary validation studies for kava quality assessment (Lebot, Michalet & Legendre, 2019; Liu et al., 2018), and provides a representative basis for method evaluation. Additional samples were collected from routine daily deliveries to ensure representativeness of commonly traded kava products. The dried samples were chopped into pieces separately (roots/chips), grounded, and sieved at room temperature to obtain particle smaller than two mm. The sieved material was homogenized, and stored in duplicate in ziplock (Thermo Fisher; Waltham, MA, USA) bags. All samples for moisture determination were weight using an analytical balance with a resolution of 0.001 g.

Moisture content was determined using an ISO-certified infrared (IR) moisture analyzer (MB120, Ohaus), which measures weight loss during thermal drying while detecting water absorbance with an IR sensor. The IR (halogen) moisture analyzer was calibrated prior to analysis. Mass calibration was verified using traceable calibration weights, and temperature performance of the heating unit was checked using the manufacturer’s temperature calibration procedure. For moisture using the IR moisture analyzer, 5 g of pulverized kava sample was placed in the sample tray and heated at 105 °C. The instrument automatically terminated the drying process once a constant weight (<1 mg change over 30 s) was achieved. Analyses were performed in triplicate for each of the ten sub-samples, and the final moisture content was recorded directly from the analyzer display.

For moisture oven method, the oven temperature was verified using a calibrated laboratory oven with traceable temperature calibration. Moisture measurement was performed using an analytical balance with a resolution of 0.001 g. Oven drying was conducted in a forced-air laboratory oven (Thermo Fisher Scientific Inc., Waltham, MA, USA) at 105 °C. The mass measurements were performed using a calibrated analytical balance with calibration traceable to national standards. Instrument performance was verified prior to analysis. Another set of sub-samples (5 g each) from the same kava chips and roots were analyzed for moisture using the oven-drying method of AOAC at temperature of 105 °C, with drying times of 0.5 h, 1.5 h, and 2 h. After drying, samples were placed in a desiccator containing regenerated silica gel as a desiccant and allowed to cool to room temperature before consecutive weighing to constant weight. This prevents absorption of atmospheric moisture and ensures accurate determination of the sample’s dry weight. According to AOAC, constant weight is reached when repeated weighing after drying shows little (≤ 0.001 g) or no change in sample weight between consecutive weighing, indicating complete moisture removal (Thiex, 2009). The data collected were used to establish a relationship between drying time and the IR method. The accuracy, recovery and linearity were calculated based on these results.

An additional kava powder samples of the same collection was prepared and tested for moisture content at four different time points to assess repeatability and reproducibility. In five trials, the same kava sample was tested under identical conditions for each time point to determine repeatability. To evaluate reproducibility, the same kava sample was analyzed every 5 days.

A sample of five kava powder was used to determine specificity and selectivity of the oven-drying method *via* Fourier Transform Infrared (FTIR) Spectroscopy (Bruker’s Alpha II, Germany). Duplicates of each of the five kava powder samples were oven-dried at 1 h intervals, with spectral data collected after cooling at each time point. FTIR spectra were acquired in absorbance mode over the range 4,000–400 cm^−^^1^ with a resolution of two cm^−^^1^ and 23 scans per sample. The spectral distributions obtained were analyzed to evaluate chemo-type variation over drying process time. Principal Component Analysis (PCA) with auto-scaling was applied to FTIR spectral data obtained from kava powder samples which were oven-dried at hourly intervals (0.5 h, 1 h, 1.5 h, and 2 h). The spectral data pre-processed using baseline correction and normalization, followed by visualization of clustering patterns. Grouping in the PCA score plots was based on drying time intervals, permitting assess to chemical profile changes in kava samples over time. The grouping method enables the identification of trends in spectral variations corresponding to moisture loss and chemical transformations during drying process.

### Effect of drying on colorimetric absorbance

A set of five kava powder samples were randomly collected from samples delivered for routine quality check and were mixed in a food mixer to ensure homogeneity prior to analysis. The prepared samples were dried in the oven (Thermo Fisher; Waltham, MA, USA) at various temperatures; 105 °C (reference temperature), 115 °C, 120 °C, 125 °C, and 130 °C in duplicate for 4 h. Absorbance was recorded following the colorimetric test procedure; drying continued, until constant weight was reached or up to 4 h to determine the drying endpoint. To ensure reliable colorimetric measurements, solvent control was included. Acetone, used as the extraction solvent, served as the blank sample to establish a baseline absorbance. No separate negative controls were used. This approach allows differentiation between the true sample signal and any absorbance contributed by the solvent itself. Dried kava sample stability was assessed using accelerated life testing. Shadrack (2024) indicates that samples with 12% moisture may remain stable for only 3 months at room temperature, while lower-moisture samples can last up to 1 year. In this study, all samples were analyzed within 1 month while stored at room temperature.

### Weighing and drying condition

An average of 5 g of each sample was accurately weighed using an analytical balance and placed in clean, dry pre-weighing petri dishes. Samples were dried in a forced-air convection (Thermo Fisher Scientific Inc., Waltham, MA, USA) oven at 105 °C according to AOAC guidelines. After each drying interval, samples were removed and allowed to cool to room temperature in a desiccator containing fresh desiccant to prevent moisture uptake before weighing. Drying and weighing were repeated until constant weight was achieved, defined as a mass change of less than 0.001 g between successive measurements.

### Statistical analysis

The kava quality trend data from 2016 to 2024 was organized and analyzed using analysis of variance (ANOVA) with two-sample t-tests to examine the relationships based on least square (LS) means, utilizing Minitab 19 software (Minitab LLC, State College, PA, USA). The XLSTAT 2018 software (Addinsoft, Paris, France) was used to test accuracy, Bland Altman method agreement, recovery, precision (repeatability and reproducibility) and effect of weight loss on absorbance using ANOVA. The LS means were reported due to unequal sample sizes. The Linearity, specificity and selectivity were analyzed using Origin Pro 2014 software (Origin Lab Cooperation, Northhampton, MA, USA). The correlation between absorbance and weight loss was analyzed using the analysis ToolPak in Microsoft Excel 2019 software.

## Results

### Kava quality by colorimetric method

**Figure 1:**
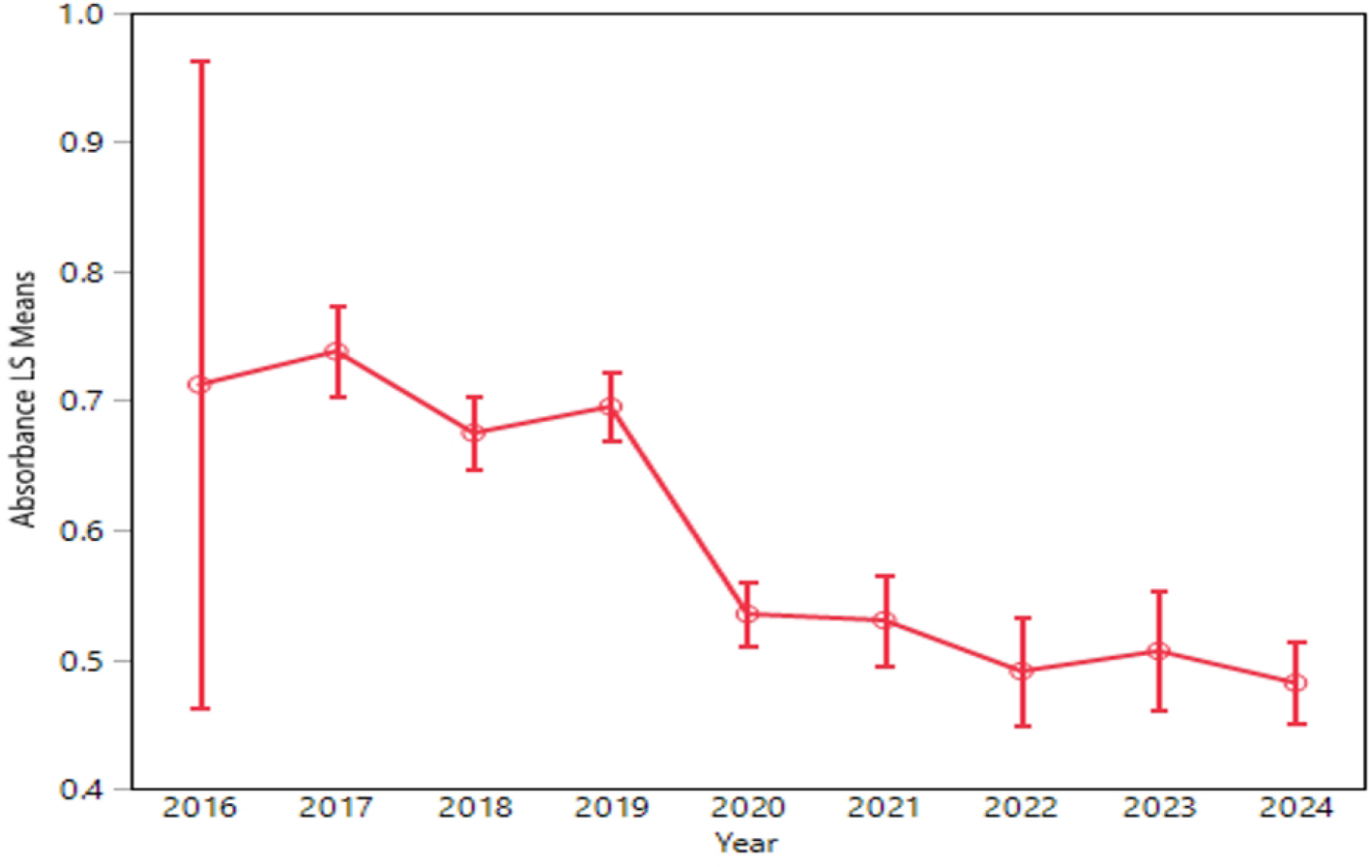
Dry kava quality trend for Vanuatu from 2016 to 2024. Least square means (± standard error) of colorimetric absorbance of the acetone extract of dry kava.

The colorimetric absorbance of Vanuatu dry kava exports from the year 2016 to 2024 in Fig. 1 shows a declining trend. The kava absorbance data from 2016 to 2024 were obtained from samples collected by the Vanuatu Bureau of Standards (VBS) through its routine quality monitoring program. The samples originated from registered kava exporters across different provinces of Vanuatu and were analyzed following the national kava quality testing protocol. A total of 2,838 tests were conducted on 2,838 unique kava samples during this period. The comparative results consistently for year 2016 showed the LS mean absorbance was around 0.72 which is below the 0.84 nobility threshold range, confirming that most samples tested correspond to noble kava types suitable for export. A notable drop occurred between 2019 and 2020, suggesting a possible change in kava composition, processing, or variety exported during or after 2019.

Table 1 shows comparative two sample t-tests of absorbance by year relative to the overall mean or intercept (baseline). The year 2017 to 2024, a statistical significance was observed compared to the baseline. The year 2017 and 2019 have negative estimates and *p* < 0.001, indicating a significantly higher absorbance than the baseline. In 2020 inward, the estimates become positive, indicating lower absorbance relative to the baseline. The differences between absorbance remain statistically significant. The most significant decrease is seen in 2024 with an estimate of −0.11 and *p* < 0.001.

**Table 1:** Comparative analysis of kava extracts colorimetric data using two sample t-test from 2016 to 2024.

**Term**	**Scaled estimate**		**Std error**	**t ratio**	**Prob>—t—**
Intercept	0.60	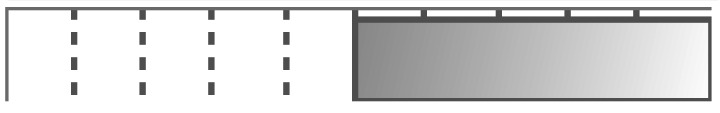	0.02	39.27	<.0001^*^
Year 2016	0.12	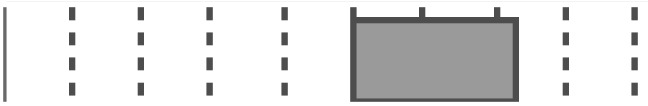	0.11	1.03	0.3051
Year 2017	0.14	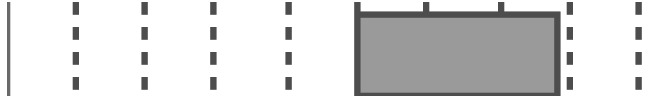	0.02	6.49	<.0001^*^
Year 2018	0.08	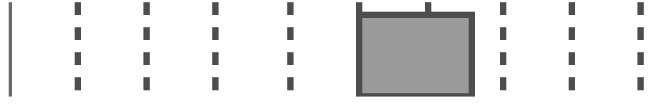	0.02	4.03	<.0001^*^
Year 2019	0.10	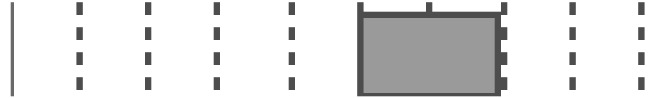	0.02	5.19	<.0001^*^
Year 2020	−0.06	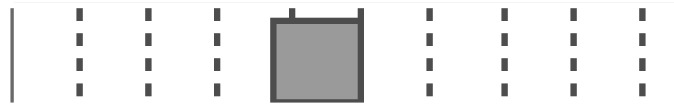	0.02	−3.23	0.0013^*^
Year 2021	−0.07	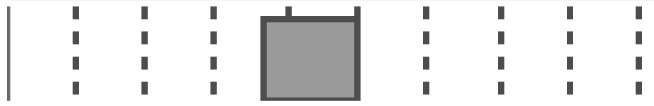	0.02	−3.01	0.0027^*^
Year 2022	−0.11	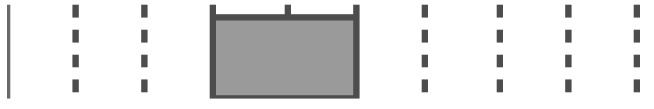	0.02	−4.40	<.0001^*^
Year 2023	−0.09	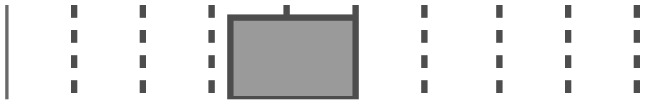	0.03	−3.50	0.0005^*^
Year 2024	−0.11	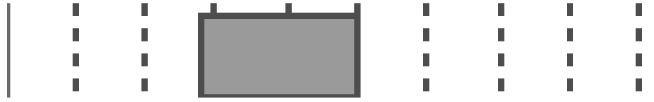	0.02	−5.53	<.0001^*^

**Notes.**

* The asterisks (*) indicated significant differences in colorimetric absorbance mean of kava extract for each year compared to 2016.

### Validation of moisture content in kava using oven-drying method (AOAC @ 105 °C)

#### Accuracy

Table 2 showed accuracy of the oven method for every hour compared to the IR moisture method. It is evident that the 2 h oven-drying method is strongly related to the IR method with high precision and accuracy compared to the rest of the oven method with the lowest residual mean square error (RMSE).

**Table 2:** Accuracy of the oven method with time compared to IR method.

**Drying time (hour)**	**R** ^**2**^ ** (Accuracy)**	**Bias (% Mean Diff)**	**Std Dev (% Precision)**	**RMSE**
0.5	0.97	−0.20	0.29	0.35
1	0.98	0.01	0.25	0.25
1.5	0.97	0.03	0.31	0.31
2	0.99	0.12	0.20	0.20

**Figure 2:**
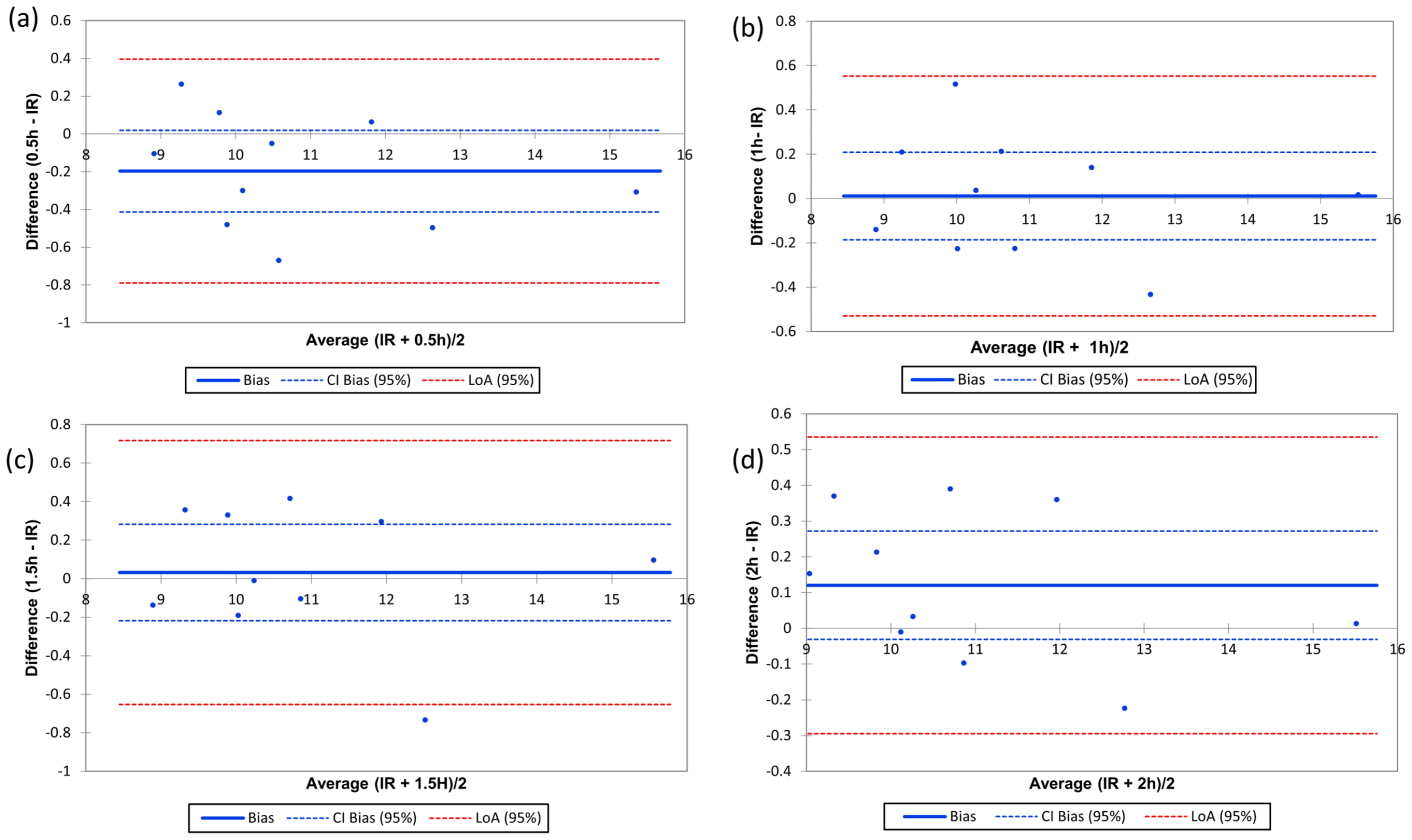
Limits of agreement between the AOAC oven-drying method at 105 °C and the infrared (IR) moisture. Bland–Altman plots showing the limits of agreement between the AOAC oven-drying method at 105 °C and the infrared (IR) moisture method for different drying times (0.5 h, 1 h, 1.5 h, and 2 h). The solid blue line indicates the mean bias, while the dashed red lines represent the limits of agreement (mean difference ± 1.96 SD).

The Bland-Altman analysis is employed in this study to assess the two quantitative methods (Giavarina, 2015), bias refers to the mean difference between the two measurements methods in percentage (%) moisture. Limits of agreement (LoA) provides information on the range that most differences between the two methods are expected to fall (Sedgwick, 2013). The LoA between the IR moisture analyzer and the AOAC oven-drying method at 105 ^∘^C was assessed using Bland–Altman analysis across four oven drying durations (0.5 h, 1 h, 1.5 h, and 2 h) shown in Fig. 2. The bias decreased with increasing drying time, indicating convergence of the IR method with the oven method. At 2 h, the bias was minimal, and 95% of the differences fell within narrow limits of agreement (± 0.25% moisture), suggesting that 2 h oven drying provides the best reference point for validating IR results. No significant trend in proportional bias was observed across the range of values. These findings support the suitability of the IR method as a rapid alternative when properly calibrated against a 2 h oven standard.

#### Recovery

Table 3 shows moisture recovery between the two methods varied with oven-drying time, reaching a maximum at 2 h, where the lowest percentage deviation was also observed.

#### Linearity

**Figure 3:**
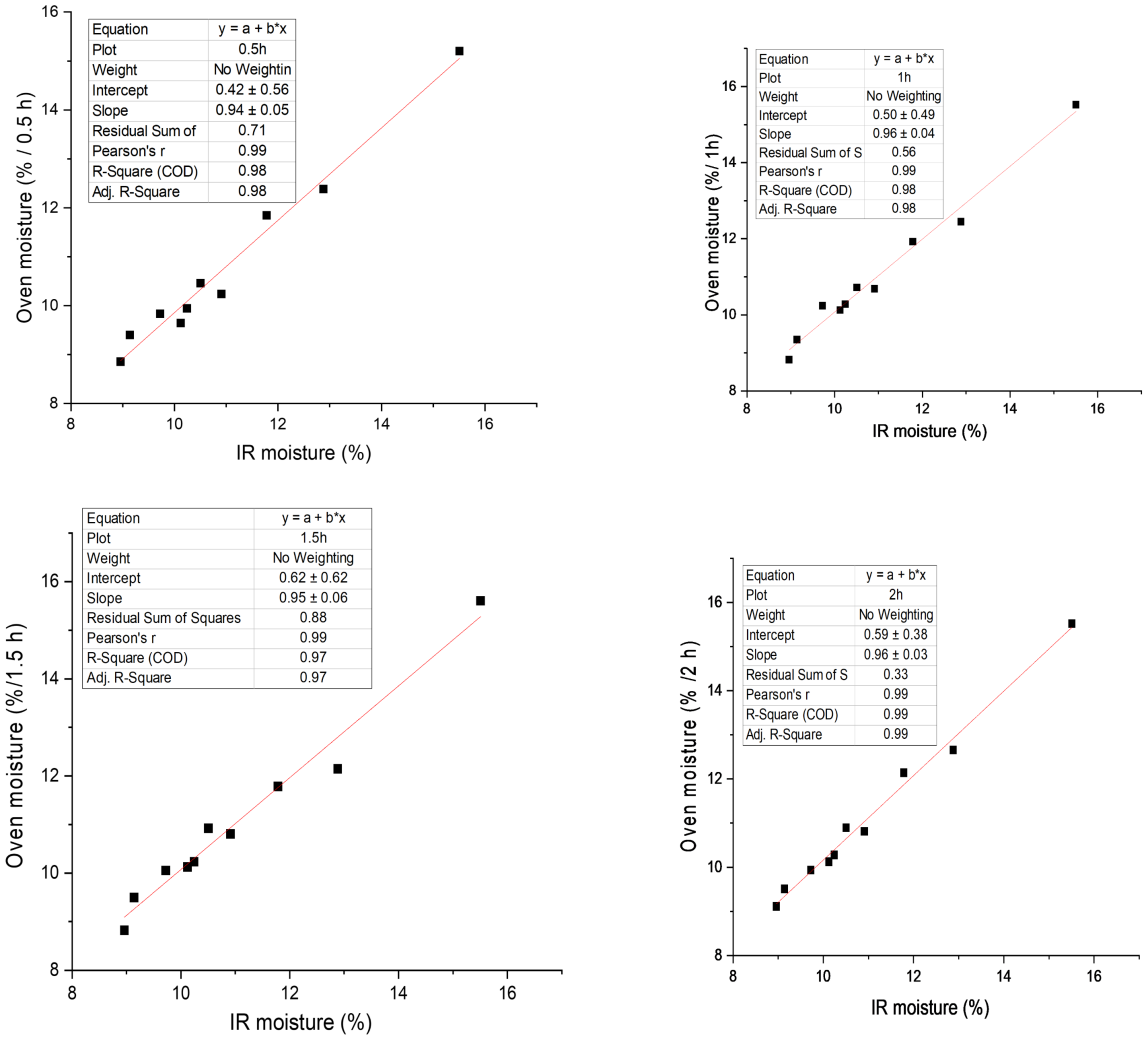
Linearity between IR moisture method and oven method with time. The linearity of moisture content in dry kava samples using AOAC oven drying method at 0.5, 1, 1.5 and 2 h drying time compared to IR method. The model performance is evaluated using R-Square coefficient.

Figure 3 showed the linearity between IR moisture method and oven method with time (h), in four drying time points. Although the other time point in drying showed strong linearity between methods, the IR moisture content at time of 2 h drying has the strongest linearity coefficient (*R*^2^ = 0.99).

#### Specificity/Selectivity

**Figure 4:**
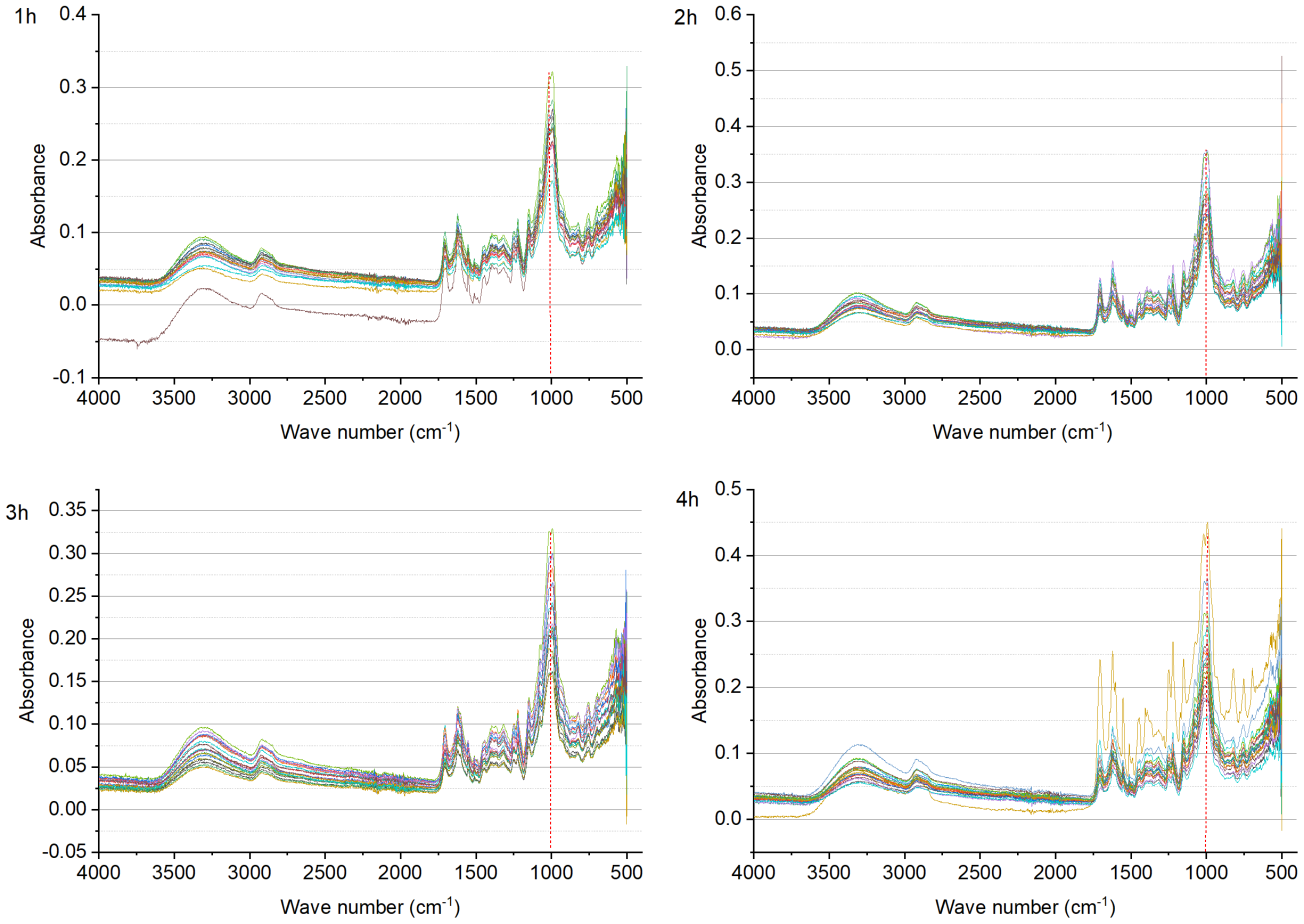
Specificity evaluation of the oven moisture method using FTIR spectral distribution of dried kava sample with drying time. Spectral distribution of dried kava sample for every 1 hr to 4 hr of drying. The vertical red dashed line indicates changes in carbohydrade region at 1,050 cm^−1^.

Figure 4 showed the FTIR spectra of kava sample during oven heating at 105 °C, showing absorbance over time in 4 hrs. The red dashed lines (1,050 cm^−1^) indicate regions (carbohydrate or polysaccharides) where the absorbance decreases most significantly which are potential qualitative indicators of compound loss after initial moisture removal.

**Figure 5:**
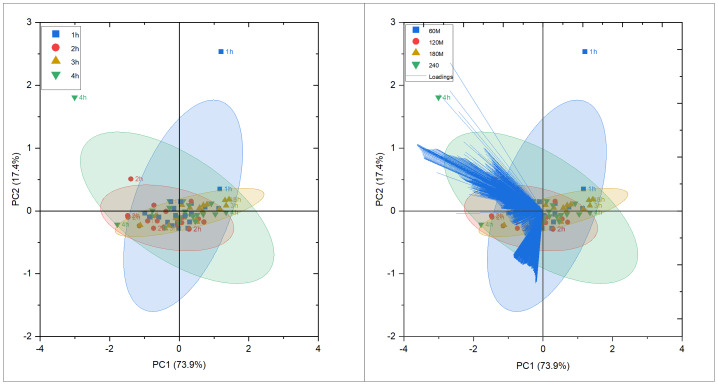
Principal component analysis plot (A) and biplot (B) for dry kava moisture with temperature. PCA plot for kava moisture by oven moisture method from 1 to 4 h drying time. (A) score plot for each group, (B) biplot or loading plot for each group. Blue represent 1 h, red represent 2 h, orange represent 3 h and green represent 4 h drying time.

The principal component analysis (PCA) grouping based on the sample spectral data for 1 h, 2 h, 3 h and 4 h drying time at 105 °C is shown in Fig. 5 where PC1 explaining 73.9% of the variation and PC2 explaining 17.4%. Altogether, the PC plots accounting for over 91% of the total variability, which means the plot gives a strong representation of the data structure. The best lowest spectral variation was observed at 2 h and 3 h of drying time demonstrating that this time points could be used as reference time due to spectral stability. The 1 h and 4 h drying time showed high instability of chemo-type due to high variation.

**Table 3:** Moisture recovery for dry kava using AOAC oven method compared to the IR method.

**Drying time (hour)**	**Mean recovery (%)**	**Std Dev (%)**
0.5	98.33	2.67
1	100.43	2.35
1.5	100.36	2.78
2	101.25	1.88

The FTIR spectral regions that are affected during heating for moisture is shown in Table 4. These wavenumber regions suggest that after moisture loss, esters, lipids, and volatile alcohols may be further evaporating or degrading at 105 °C. This suggest that further heating at 105 °C appears to affect esters, lipids, and alcohol-containing compounds. This finding illustrates the thermal degradation relevance for understanding compound stability in kava during drying.

#### Precision

Table 5 showed precision is excellent across all four drying times. The RSD (%) for repeatability is below 1% for all meaning that all measurement is very consistent. The reproducibility RSD (%) is also low (≤ 0.60%) and is stable across days. Moisture increases slightly with drying time, suggesting longer drying (90–120 min) gives a more complete moisture release. This mean that the drying time of 1.5 h to 2 h at 105 °C is ideal for complete moisture determination.

### Effect of drying condition on kava quality

Table 6 showed relationship between moisture content and absorbance after drying. It is clearly observed that there is significant linear relationship between moisture content and absorbance of dry kava.

**Figure 6:**
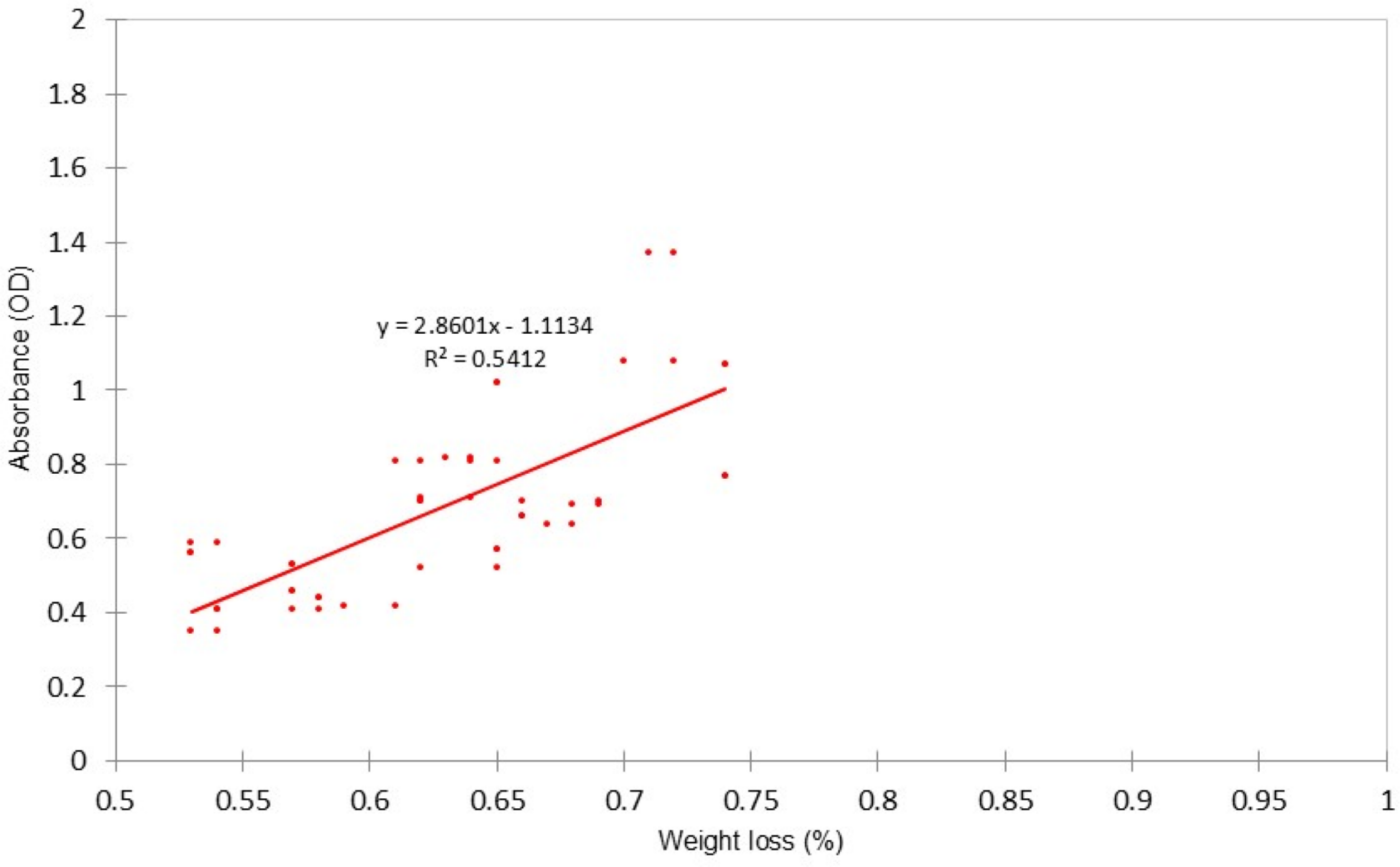
Relationship between absorbance of kava extract and weight loss. Correlation between colorimetric absorbance at 440 nm of kava extract and weight loss after drying at 105 °C, 115 °C, 120 °C, 125 °C and 131 °C in 4 h.

The moisture content at 4 temperatures of drying (105 °C, 115 °C, 120 °C, 125 °C and 131 °C in 4 h) and the relationship with colorimetric absorbance using acetone as solvent is shown in Fig. 6. It is clearly observed that as weight loss increases, absorbance increases even at very small percentage of change in weight loss. This revealed a strong linear relationship with an R^2^ coefficient of 0.54.

**Figure 7:**
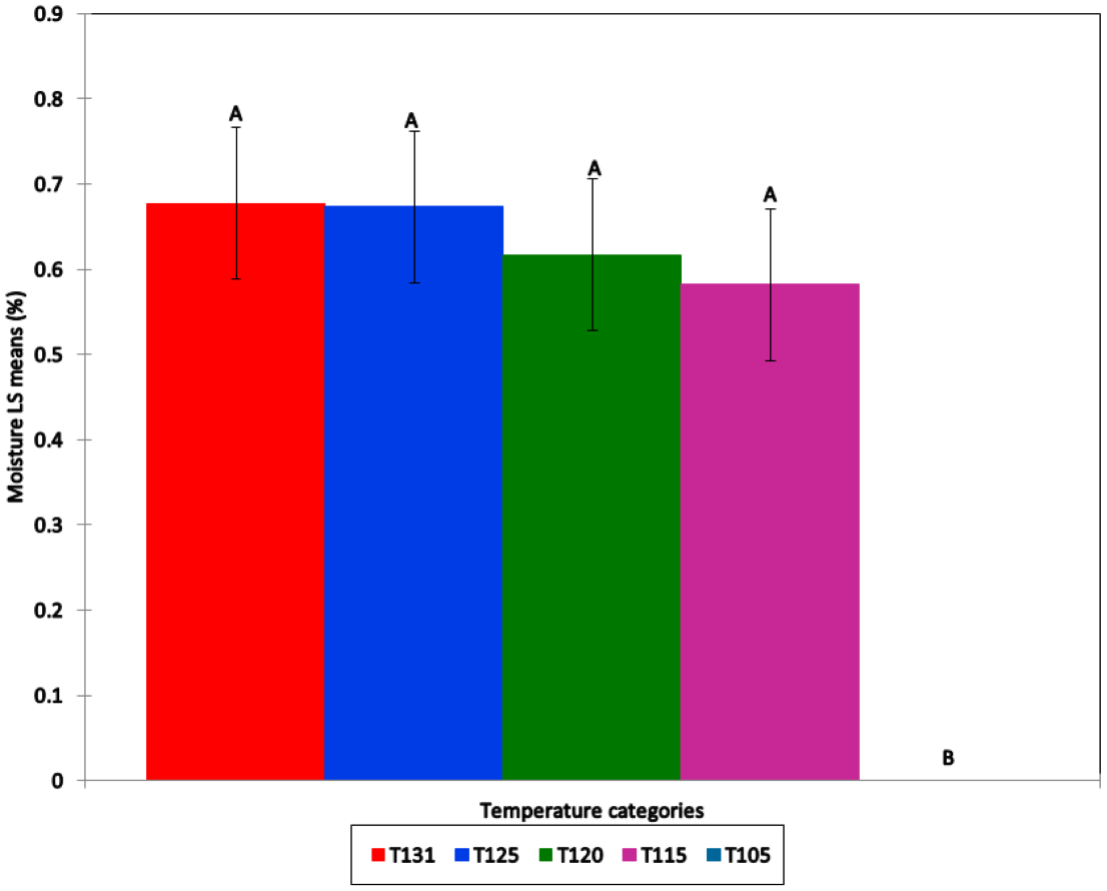
Effect of drying temperature on weight loss of dry kava. The effect of drying temperature on weight loss with temperature over 4 h of drying. Different letters indicate significant differences between means (*p* ≤ 0.05).

The effect of drying temperature on weight loss of dry kava in 4 h drying time is shown in Fig. 7. The LS means of moisture content (%) at 105 °C (baseline) is significantly different from those at drying temperatures of 115 °C, 120 °C, 125 °C and 131 °C. A constant weight is observed at temperatures 125 °C and 131 °C in 4 h of drying with a weight difference of 0.004%.

**Table 4:** Wave number and functional group of compounds alteration in kava with drying.

**Wave number (cm-1)**	**Functional group**	**Compound type**	**Interpretation**
1,740	C=O stretch	Esters, aldehydes, ketones	Loss of volatile esters or lipid oxidation products
2,920	C-H stretch (asym)	Alkanes (from fatty acids/oils)	Evaporation or thermal breakdown of lipid chains
1,640	C=O stretch/H-O-H bend	Alkenes, water	Decrease in unsaturated compounds or traces of water
1,050	C-O stretch	Alcohols, ethers, polysaccharides	Loss of polar compounds or breakdown of carbohydrate content or sugar

**Notes.**

* Band assignments were based in comparison with standard literature references of Pavia et al. (2001) and Berthomieu & Hienerwadel (2009).

**Table 5:** Repeatability and reproducibility of AOAC moisture method over 4 time points of oven drying.

**Drying time (hour)**	**Repeatability (mean %)**	**SD**	**% RSD**	**Reproducibility (mean %)**	**SD**	**% RSD**
**0.5**	12.61	0.13	1.00	12.60	0.08	0.60
**1**	12.60	0.10	0.77	12.59	0.07	0.58
**1.5**	12.73	0.05	0.39	12.72	0.08	0.59
**2**	12.84	0.03	0.22	12.80	0.04	0.29

**Table 6:** Pearson’s correlation coefficient of the relationship between moisture and absorbance using oven method.

**Variables**	**Moisture (%)**	**Absorbance after drying**
**Moisture (%)**	0	<0.0001
**Absorbance after drying**	<0.0001	0

## Discussion

The quality assessment data reveal a notable improvement in the chemical quality of Vanuatu dry kava exports over the period from 2016 to 2024, as evidenced by a consistent decline in colorimetric absorbance values (Fig. 1). The decrease in absorbance from 0.72 in 2016 to 0.48 in 2024 signifies a shift towards noble kava varieties and improved post-harvest processing (Table 1). Notably, the most substantial and statistically significant declines occurred after 2019. These trends correlated with regulatory and quality initiatives following the implementation of the Vanuatu Kava Act of 2002, suggesting increased adherence to standards and better selection of noble and non-noble cultivar (Lindstrom, 2009). These observations align with the findings of previous studies where cultivar selection is a key determinant of flavokavain levels (Lebot, Do & Legendre, 2014; Liu et al., 2018). Additionally, flavokavain concentrations are anticipated to be higher in extracts absorbance prepared using organic solvent-based methods compared to water-based extraction. This is because flavokavains are insoluble in water and tend to be present in low concentrations in noble kava varieties (Lebot, Kaoh & Legendre, 2020).

In this context, lower absorbance values are indicative of higher-quality kava, and is related to low concentrations of undesirable or non-kava lactone compounds such as polyphenols, flavonoids, and other oxidisable materials that can affect the purity, safety, and psychoactive consistency of kava products (Lebot, Kaoh & Legendre, 2020). Elevated absorbance often correlates with poor-quality cultivars (*e.g.*, tudei or wild kava), suboptimal post-harvest processing, or excessive plant fibre content, all of which diminish the desirability and export value of kava (Lebot & Legendre, 2016).

This could have reduced the presence of unwanted secondary metabolites highly present in tudei kava (Cheung et al., 2022; Pluskal et al., 2019). Better post-harvest handling by standardization of drying, peeling, and storage practices may have limited the oxidation of plant tissues and minimized extractable impurities. The enhanced quality control measures such as the Vanuatu Kava act, quality control checks and market-driven incentives have also encouraged exporters to adopt stricter quality benchmarks aligned with international safety and efficacy standards. In addition, the increased awareness and training of local growers and processors may improve their knowledge on quality parameters, promoting better assessments regarding harvesting maturity and material handling (Gerren, 2017).

While these improvements are commendable, it is essential to continue monitoring trends and ensuring consistency in kava quality. The year-on-year statistical significance, particularly from 2020 to 2024 (*p* < 0.01), underscores a sustained reduction in unwanted compounds, enhancing the overall safety and appeal of Vanuatu’s kava in global markets. There is a shortage in quantity of dry kava export in 2024–2025 fiscal year, suggesting a need for strict observation from quality test data to prevent poor quality kava from entering international market. In such situation, it is important to maintain and enforce quality-based export standards that reward lower absorbance profiles, support cultivar mapping and traceability. This ensures noble varieties dominate exports, increase the need for continual training of farmers and processors on quality-linked practices, and institutionalize regular analytical monitoring (*e.g.*, FTIR or colorimetric screening) as part of routine quality control.

In validating the oven-drying method, results showed that drying at 105 °C for 2 h yielded the most accurate and precise moisture content measurements when compared to the IR method. The high linearity (*R*^2^ = 0.99), low RMSE (0.20), and tight limits of agreement (Bias = 0.12%, LoA = ±0.25%) establish the 2 h oven-drying method as a standard reference for routine quality control. These validation results align with AOAC guidelines and are supported by similar outcomes in food moisture analysis studies (Park & Bell, 2004; Thiex, 2009). Recovery rates further confirmed the effectiveness of the 2 h oven-drying method, showing the highest moisture recovery (101.25%) and lowest variability (SD = 1.88%) among all tested time points. Moreover, repeatability and reproducibility were excellent at this drying time, with RSD values below 1%, which meets international precision standards (Reh et al., 2006; Fricke & Chapman, 2017).

FTIR spectral analysis provided further information into compound stability during drying as a qualitative approach (Figs. 4 & 5) while the degree of change in same sample was measured with the colorimetric absorbance with drying temperature. The significant absorbance loss at 1,050 cm^−^^1^, corresponding to carbohydrates and polysaccharides, indicated the volatilization or degradation of polar compounds after initial moisture removal. The PCA results highlighted moisture stability at 2 h and 3 h drying durations, suggesting optimal balance between drying and compound preservation. Importantly, the correlation between moisture content and colorimetric absorbance was statistically significant (*p* < 0.001), confirming that moisture variations can distort absorbance readings. This results also suggested that processing, chemical composition, and varietal differences could also contribute to moisture loss variability. This finding validates concerns raised by Lhuissier et al. (2017) and supports the necessity of moisture correction for reliable quality classification.

The impact of drying temperature was also evident. While moisture plateaued at higher temperatures (125 °C and 131 °C) with weight difference of 0.004%, extended drying led to degradation of key compounds. In reference to AOAC, constant weight specific time intervals and weight differences depend on the analytical method and sample type analysed (Henshall, 2012). These results caution against over-drying and support a standardized drying time of 2 h at 105 °C to preserve moisture accuracy and chemical integrity. Furthermore, stretching vibrations (1,740 cm^−1^) of C=O were observed attributing to ketones formed from degradation of kavalactone and various phytochemicals. In addition, stretching bands (3,200–3,600 cm^−1^) indicates elevated moisture re-absorption. The spectral changes show that excessive drying reduces moisture and alters the chemical profile of kava, it is therefore important to control drying parameters. Collectively, these findings provide strong evidence that the 2 h oven-drying method at 105 °C is both scientifically validated and operationally practical for determining moisture content in kava. The method accurately quantifies moisture to support microbial risk control; however, further evaluation of the chemical profile particularly kavalactone and flavokavain content is needed to confirm the preservation of noble kava quality.

Other limitations of this analysis is the uncontrolled sources of variation likely due to the heterogeneity related to kava variety, post-harvest processing practices (*e.g.*, peeling, washing, drying), and storage conditions prior to analysis which was not controlled, as samples were submitted by exporters for routine quality assessment rather than collected under a standardized experimental design. These factors can independently influence spectral characteristics and absorbance values, increasing unexplained variance while still allowing detection of meaningful qualitative differences relevant to export quality assessment. The small sample size for the method comparison and the lack of reference and blank kava sample to assess method performance is also a limitation.

While this study focused on physical and spectral (FTIR) characteristics, future research will incorporate comprehensive chemical profiling and quantitative assays to better establish the relationship between drying conditions and export-grade quality. Overall, adopting this method could enhance the reliability of routine quality assessments, minimize dependence on costly instrumentation, and strengthen regulatory compliance across the kava value chain.

## Conclusion

This study demonstrates that kava quality in Vanuatu has improved significantly between 2016 and 2024, supported by declining colorimetric absorbance values and the implementation of stricter quality control measures. The validated 2 h oven-drying method at 105 °C proved to be a practical and reliable approach for determining moisture content, showing strong agreement with infrared moisture analysis. Qualitatively, FTIR analysis confirmed chemical changes during drying, emphasizing the importance of standardized drying conditions. A key finding was the strong correlation between % moisture content and absorbance, highlighting the need for future research to develop a moisture-based absorbance correction model to improve the accuracy of noble cultivar classification based on colorimetric analysis and ensure more consistent export quality assessments.

However, this study is subject to several limitations. Validation was conducted on a limited sample set, which may constrain statistical generalization. Geographic variability and differences in processing practices across regions were not fully captured, and samples were submitted by exporters rather than systematically collected at origin. In addition, the absence of comprehensive chemical profiling limits direct linkage between absorbance values and specific chemical markers of quality or nobility. Future study should expand the scope of the oven-drying method validation across different kava varieties, regions, and processing conditions will strengthen its applicability. The developed method may serve as a national reference after additional validation. This will protect Vanuatu’s identity in global markets and may contribute to the economic viability of kava industry.

##  Supplemental Information

10.7717/peerj.21102/supp-1 Supplemental Information 1 Effects of drying temperature on absorbance

10.7717/peerj.21102/supp-2 Supplemental Information 2 Original comparative analysis of kava samples with AOAC oven method and infrared cobustion method

10.7717/peerj.21102/supp-3 Supplemental Information 3 Raw Data

## Additional Information and Declarations

### Data Availability

The following information was supplied regarding data availability: The raw data is available in the Supplemental Files.
